# Comparison of Pathogen DNA Isolation Methods from Large Volumes of Whole Blood to Improve Molecular Diagnosis of Bloodstream Infections

**DOI:** 10.1371/journal.pone.0072349

**Published:** 2013-08-15

**Authors:** Anne J. M. Loonen, Martine P. Bos, Bart van Meerbergen, Sigi Neerken, Arnold Catsburg, Irene Dobbelaer, Roel Penterman, Geert Maertens, Paul van de Wiel, Paul Savelkoul, Adriaan J. C. van den Brule

**Affiliations:** 1 Jeroen Bosch Hospital, Department of Molecular Diagnostics, 's-Hertogenbosch, The Netherlands; 2 Fontys University of Applied Sciences, Department of Medical Molecular Diagnostics, Eindhoven, The Netherlands; 3 VU University Medical Center, Department of Medical Microbiology and Infection Control, Amsterdam and Microbiome Ltd., Houten, The Netherlands; 4 Biocartis NV, Mechelen, Belgium; 5 Philips Research, Department of Molecular Diagnostics, Eindhoven, The Netherlands; University of Houston, United States of America

## Abstract

For patients suffering from bloodstream infections (BSI) molecular diagnostics from whole blood holds promise to provide fast and adequate treatment. However, this approach is hampered by the need of large blood volumes. Three methods for pathogen DNA isolation from whole blood were compared, i.e. an enzymatic method (MolYsis, 1–5 ml), the novel non-enzymatic procedure (Polaris, 1–5 ml), and a method that does not entail removal of human DNA (Triton-Tris-EDTA EasyMAG, 200 µl). These methods were evaluated by processing blood spiked with 0–1000 CFU/ml of *Staphylococcus aureus*, *Pseudomonas aeruginosa* and *Candida albicans.* Downstream detection was performed with real-time PCR assays. Polaris and MolYsis processing followed by real-time PCRs enabled pathogen detection at clinically relevant concentrations of 1–10 CFU/ml blood. By increasing sample volumes, concurrent lower cycle threshold (Ct) values were obtained at clinically relevant pathogen concentrations, demonstrating the benefit of using larger blood volumes. A 100% detection rate at a concentration of 10 CFU/ml for all tested pathogens was obtained with the Polaris enrichment, whereas comparatively lower detection rates were measured for MolYsis (50–67%) and EasyMAG (58–79%). For the samples with a concentration of 1 CFU/ml Polaris resulted in most optimal detection rates of 70–75% (MolYsis 17–50% and TTE-EasyMAG 20–36%). The Polaris method was more reproducible, less labour intensive, and faster (45 minutes (including Qiagen DNA extraction) vs. 2 hours (MolYsis)). In conclusion, Polaris and MolYsis enrichment followed by DNA isolation and real-time PCR enables reliable and sensitive detection of bacteria and fungi from 5 ml blood. With Polaris results are available within 3 hours, showing potential for improved BSI diagnostics.

## Introduction

Bloodstream infections (BSI) can be caused by a wide variety of pathogens and remain a significant cause of morbidity and mortality especially in the Intensive Care Unit [1], [2], [3]. This could be significantly improved by pathogen-tailored antibiotic and antifungal treatment [4]. This requires a fast identification of the infecting pathogen. Rapidly administered, targeted therapy is also important to reduce the risk of resistance development among pathogens. Current practice for pathogen identification in BSI consists of time-consuming (24–72 hours) blood cultures. To be able to provide fast and patient tailored treatment, identification of the pathogen should be available as soon as possible, as patients in septic shock with inappropriate treatment have significantly lower survival rates [4], [5].

Culture-independent identification techniques, such as molecular diagnostics, will shorten time to result. Pathogen levels in blood of BSI patients can be as low as 1–10 colony forming units (CFU) per ml, therefore several millilitres of blood may be required to reach clinically relevant sensitivity. This poses a problem since the amount of human DNA and haemoglobin present in such samples inhibit the pathogen-specific PCR [6]. In order to reach similar sensitivities as blood cultures, where input is in the order of 10–20 ml per blood culture set, pathogen DNA enrichment methods should precede the identification PCR.

Recently, several molecular diagnostic tests for whole blood became commercially available (SepsiTest (Molzym), MagicPlex Sepsis Real-Time Test (Seegene), VYOO (SIRS Lab), and Septi*FAST* (Roche)) and were evaluated by several independent research groups [7], [8], [9], [10], [11], [12], [13]. However, none of the abovementioned tests combines pathogen DNA enrichment with fast identification, they provide either pathogen DNA enrichment or fast sensitive detection. Only the Molzym test enables pathogen DNA enrichment based on enzymatic removal of human DNA (MolYsis) using an input volume of 1 to 5 ml whole blood [14], [15]. However, the method is labour-intensive and the use of enzymes may make this test less stable. We therefore tested and evaluated a novel non-enzymatic and more rapid pathogen DNA enrichment method for blood samples, designated Polaris.

The main goal of this study was to evaluate the Polaris method and to compare its performance to the MolYsis method and a method that does not entail removal of human DNA (Triton-Tris-EDTA - EasyMAG) [16]. These methods were compared using whole blood samples spiked with frequently recovered BSI microorganisms *Staphylococcus aureus, Pseudomonas aeruginosa* and *Candida albicans*, representing Gram-positive and Gram-negative bacteria, and a fungus, respectively.

## Materials and Methods

### Ethics Statement

In The Netherlands, healthy blood donors have to sign an informed consent form when donating blood at the Sanquin institute. In this form, medical research purposes are mentioned. Research institutes can buy this blood, and donors are anonimised. Therefore, no additional informed consent was required.

### Spiking Experiments

**Figure 1:**
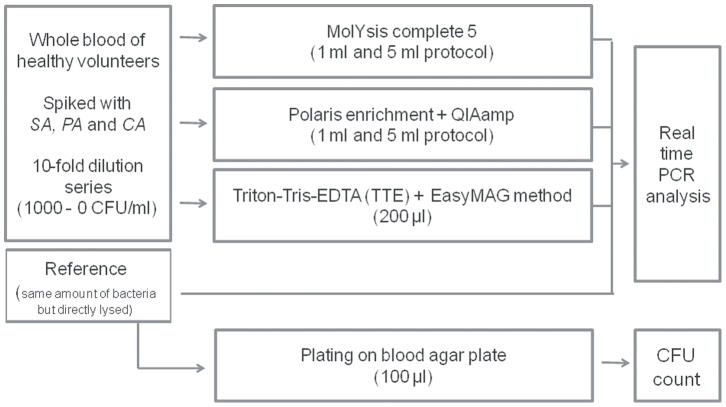
Flowchart of experimental set-up. SA, *S. aureus*; PA, *P. aeruginosa*; CA, *C. albicans*; CFU, colony forming unit.

EDTA blood from healthy human volunteers was obtained from Sanquin (bloodbank, Eindhoven, The Netherlands). *Staphylococcus aureus* (ATCC 25923), *Pseudomonas aeruginosa* (ATCC 27853), and *Candida albicans* (ATCC 90028) were used for spiking. All microorganisms were cultured overnight (O/N) on blood agar plates (TSA plates with 5% Sheep Blood, Fischer scientific, Aalst, Belgium). Subsequently, the cells were grown to mid log phase in Brain Hearth Infusion broth (*S. aureus)* or LB (*P. aeruginosa*) to ensure having a majority of actively growing cells. *C.albicans* was in mid log phase after the O/N culturing step. Hereafter, a ten-fold serial dilution was made in PBS (Merck, Darmstadt, Germany) and before spiking a live/dead staining (Life Technologies, Gent, Belgium) was performed as described by the manufacturer, to determine the ratio between live and dead pathogens (criterium used >90% living bacteria). This was performed to confirm that the majority of cells is intact since the MolYsis and Polaris method will not allow enrichment of damaged cells and free DNA. To determine the CFU per ml, 100 µl from several dilutions were plated onto blood agar plates and cultured O/N. Blood was spiked with the different dilutions, yielding 0–1000 CFU/ml blood. Reference samples consisted of similar amounts of pathogens taken from the PBS dilution series directly subjected to lysis and extraction. See Figure 1 for an overview of the experimental set-up.

### DNA Isolation Methods

Pathogen DNA was isolated with three different methods. The Triton-Tris-EDTA (TTE) pre-treatment procedure (input 200 µl blood) followed by EasyMAG isolation (BioMérieux, Marcy L’Etoile, France) was performed as described by Peters *et al.* [16]. The MolYsis complete 5 kit was used for pathogen DNA isolation from 1 and 5 ml spiked whole blood as described by the manufacturer (Molzym GmbH, Bremen, Germany).

**Figure 2:**
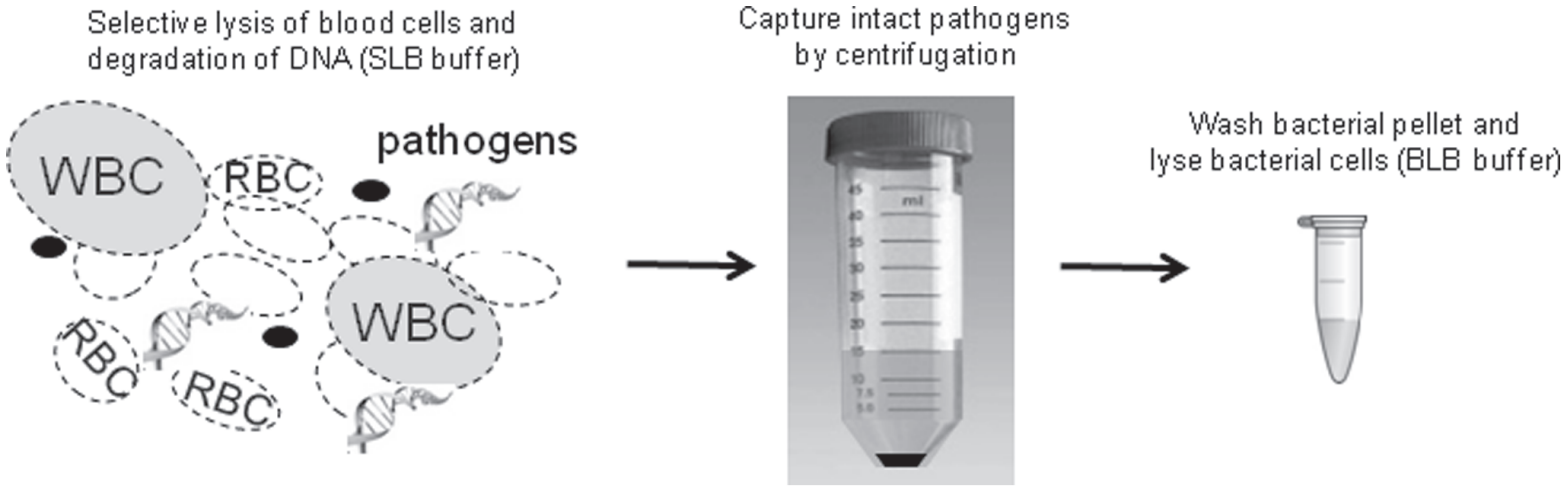
Overview of the Polaris method. Whole blood is depicted, consisting of human cells and DNA, and some pathogens. In the first step, human cells and DNA are degraded and pathogens remain intact. In the second step, intact pathogens are pelleted by centrifugation. Finally, this pellet is washed and pathogens are lysed. Subsequently, DNA can be isolated (not depicted). WBC, white blood cell; RBC, red blood cell; SLB, selective lysis buffer; BLB, bug lysis buffer.

Details of the Polaris technology (Biocartis, Mechelen, Belgium) are described elsewhere (patents WO2012168003 A1 and WO2011070507 A1). For Polaris (Figure 2), 1 or 5 ml blood was mixed with an equal volume of selective lysis buffer (SLB) for 3 minutes, to lyse blood cells and fragment the released human DNA and then 1 or 5 ml neutralization buffer was added. The selective lysis is based on a mild detergent to degrade the human cell membranes but not the bacterial and fungal cell walls. An elevated pH will ensure degradation of the released nucleic acids. Therefore, this method focuses on the enrichment of the intact bacteria and fungi from blood and not potential free pathogen DNA. The selective lysis reaction needs to be controlled in time as Gram-negative bacteria might be lysed upon prolonged exposure. Therefore an equal volume of neutralization buffer is added after 3 min. This buffer will ensure a complete arrest of the selective lysis treatment by lowering of the pH and dilution of the detergent to an ineffective concentration. At this moment in time, the pathogens will remain intact. Consecutively, suspensions were centrifuged for 15 minutes (5 ml protocol) or 10 minutes (1 ml protocol) at 2791×g. Pellets were resuspended in 1 ml washing buffer and centrifuged for 10 minutes at maximum speed in a Eppendorf centrifuge. Resulting pellets were thoroughly resuspended in 200 µl bacterial lysis buffer (BLB) and incubated for 10 minutes at 95°C on a thermomixer set at 1000 rpm. After addition of 20 µl neutralization buffer 2, lysates were further processed for DNA purification using QIAamp blood mini kit columns (Qiagen, Venlo, The Netherlands) or the generic program of the EasyMAG device.

### Real-time PCR

The RNAseP kit (Life technologies, Gent, Belgium) was used to measure the amount of human DNA. Species-specific real-time PCRs were performed to investigate the performance of each method. For detection of *S. aureus* the *tuf* gene based LightCycler 2.0 assay was used [17]. The primers and probes for detection of *S. aureus, P. aeruginosa,* and *C. albicans* are depicted in Table 1. PCR mix consisted of 12.5 µl Taqman Universal fast 2× mastermix (Applied Biosystems), 300 nM primers, 200 nM probe, and 10 µl sample (1/10 of total eluate), water was added to an end volume of 25 µl. PCRs were performed on the Biorad CFX-96 under the following conditions; 3 min 95°C followed by 50 cycles of 15 sec at 95°C and 1 min at 60°C.

**Table 1:** Overview of primers and probes used for pathogen detection.

Pathogen	gene	Forward primer	Reverse primer	Probe (FAM-BHQ1)
*P. aeruginosa*	*regA*	TGCTGGTGGCACAGGACAT	TTGTTGGTGCAGTTCCTCATTG	CCAGATGCTTTGCCTCAACGTCG
*S. aureus*	*tuf*	TCCTGGTTCAATTACACCACATACTG	GGAAATAGAATTGTGGACGATAGTTTGA	TGATAATACGTATACTTATGC
*C. albicans*	ITS-2	GGAGGGCATGCCTGTTTG	CAAGTCGTATTGCTCAACACCAA	TCGTTTCTCCCTCAAACCGCTGGG

### Statistical Analysis

For analysis of the results the Fisher’s exact test and one-way ANOVA were performed in SPSS (Version 19.0. Armonk, NY: IBM Corp). For one-way ANOVA analysis, the Bonferroni's correction for multiple comparisons was performed for comparison of the obtained Ct-values (RNAseP) for the different methods. For both statistical methods, a *p*-value less than 0.05 was considered significant.

## Results

### Performance of Polaris: Effect of Sample Volume

**Figure 3:**
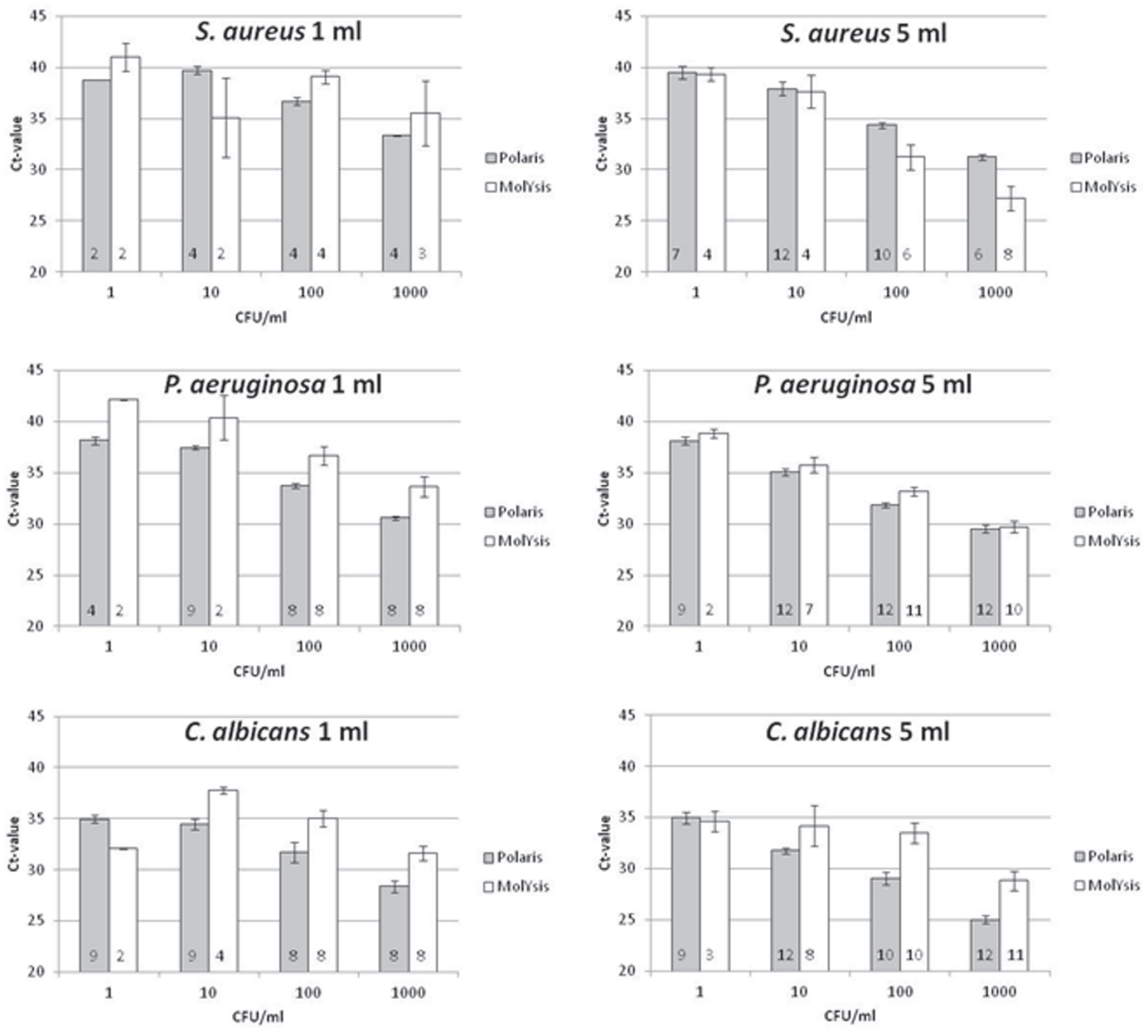
Comparison of Polaris and MolYsis methods using 1 ml and 5 ml spiked whole blood samples. The grey bars represent the Polaris samples (1 or 5 ml whole blood), and the white bars represent the MolYsis isolated samples (1 or 5 ml whole blood). SEM is shown. The numbers in the bars represent the sample numbers.

**Figure 4:**
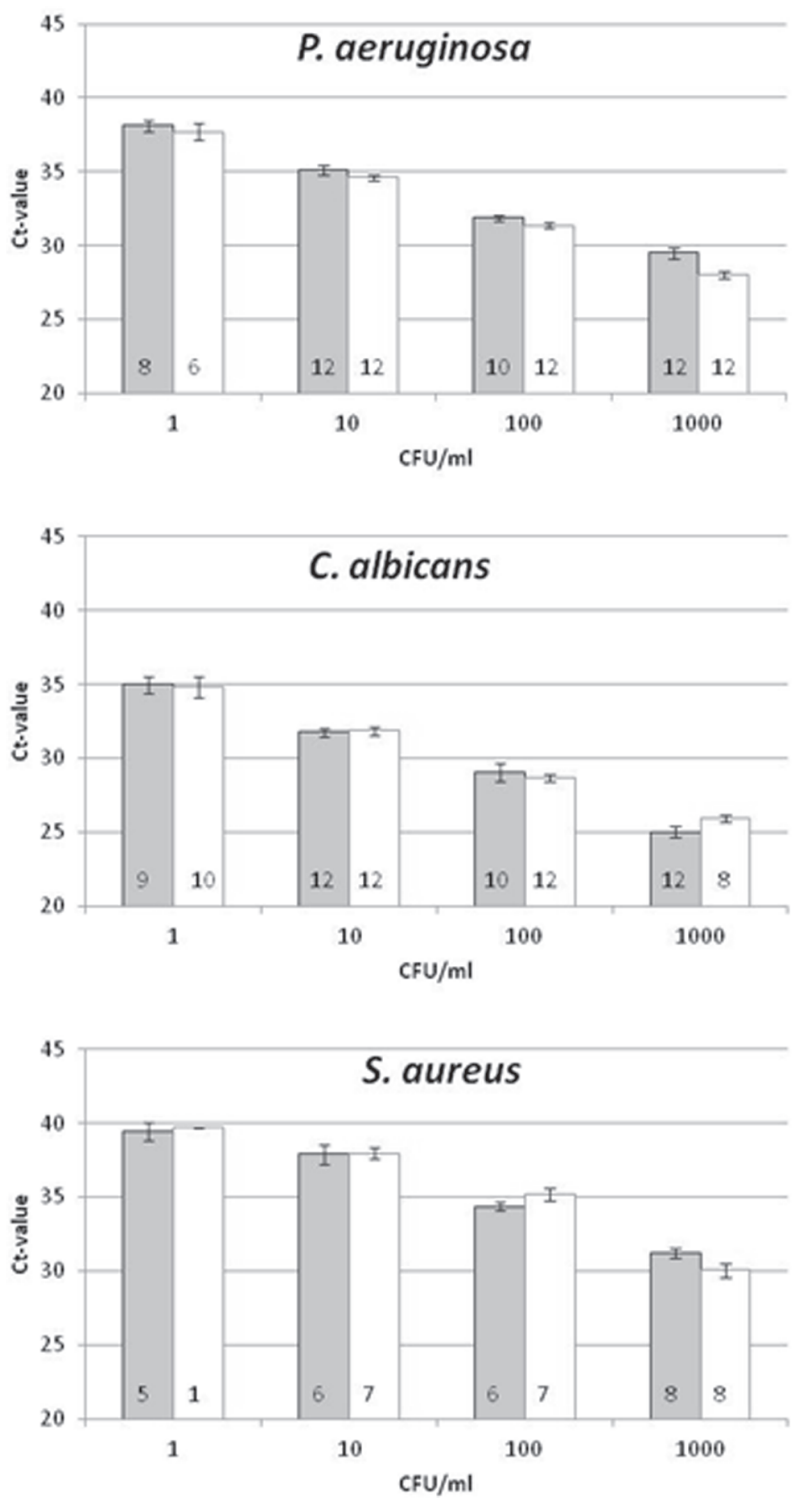
Polaris pathogen DNA isolation from reference (PBS) compared to 5 ml whole blood. The indicated pathogens were spiked in 5 ml whole blood or processed as reference samples as described in the Materials and Methods. For all pathogens similar Ct values were obtained when isolated from PBS or whole blood. The grey bars represent the spiked 5 ml whole blood samples and the white bars the reference samples. SEM is shown. The numbers in the bars represent the sample numbers.

To test the effect of sample volume on sensitivity of the Polaris procedure, a range of pathogen concentrations was spiked in 1 and 5 ml whole blood samples from healthy volunteers. Consistently lower cycle threshold (Ct) values were obtained in the PCRs when pathogen DNA enrichment was performed on 5 ml instead of 1 ml (Figure 3, grey bars (left side 1 ml, right side 5 ml)). The difference in Ct value was less pronounced in the lower ranges of pathogen concentration (1 CFU/ml). However, at this concentration a higher detection rate (*S. aureus* 12.5% (1 ml) versus 70% (5 ml); *P. aeruginosa* 44% (1 ml) versus 75% (5 ml); *C. albicans* 75% for both 1 and 5 ml) was observed for the 5 ml samples compared to those derived from 1 ml, indicating that a 5 ml sample provides a higher sensitivity than a 1 ml sample. Furthermore, the Ct-values indicate that all tested pathogens were detected with similar efficiencies (Figure 4). The selective enrichment and the pathogen lysis step perform well for the different classes of pathogens, i.e. fungal, Gram-positive and Gram-negative bacterial organisms. At the same time, this demonstrates that no pathogens are lost during the selective lysis step.

Polaris-processed spiked blood samples were compared to reference samples, containing the same amount of pathogens, but then directly lysed in BLB. At all pathogen concentrations tested, the Polaris-processed samples yielded similar Ct values as the reference samples (Figure 4), demonstrating the absence of inhibition in the blood-derived samples. All non-spiked blood samples were negative in the PCRs.

### Effect of Elution Volume in DNA Extraction

Next, it was investigated which DNA purification method following Polaris enrichment would result in optimal detection, QIAamp (elution in 100 µl EB buffer as in protocol) or EasyMAG (elution in 25 µl), followed by PCR where in both cases 10 µl eluate was used. To that end, 11 different 5 ml blood samples each containing 1 CFU/ml of *S. aureus* were processed using Polaris. Five samples were purified using the QIAamp blood mini kit and six samples were processed on the EasyMAG. Using the EasyMAG generic protocol, 5 out of 6 samples were positive in the *S. aureus* PCR. With QIAamp only 3 out of 5 samples resulted in PCR signals. These preliminary results show that the combination of Polaris and EasyMAG makes it possible to put an equivalent of 10/25×5 ml = 2 ml blood in one PCR reaction and obtain an 5/6 detection rate at a concentration of 1 CFU/ml.

### Human DNA is Efficiently Removed

**Figure 5:**
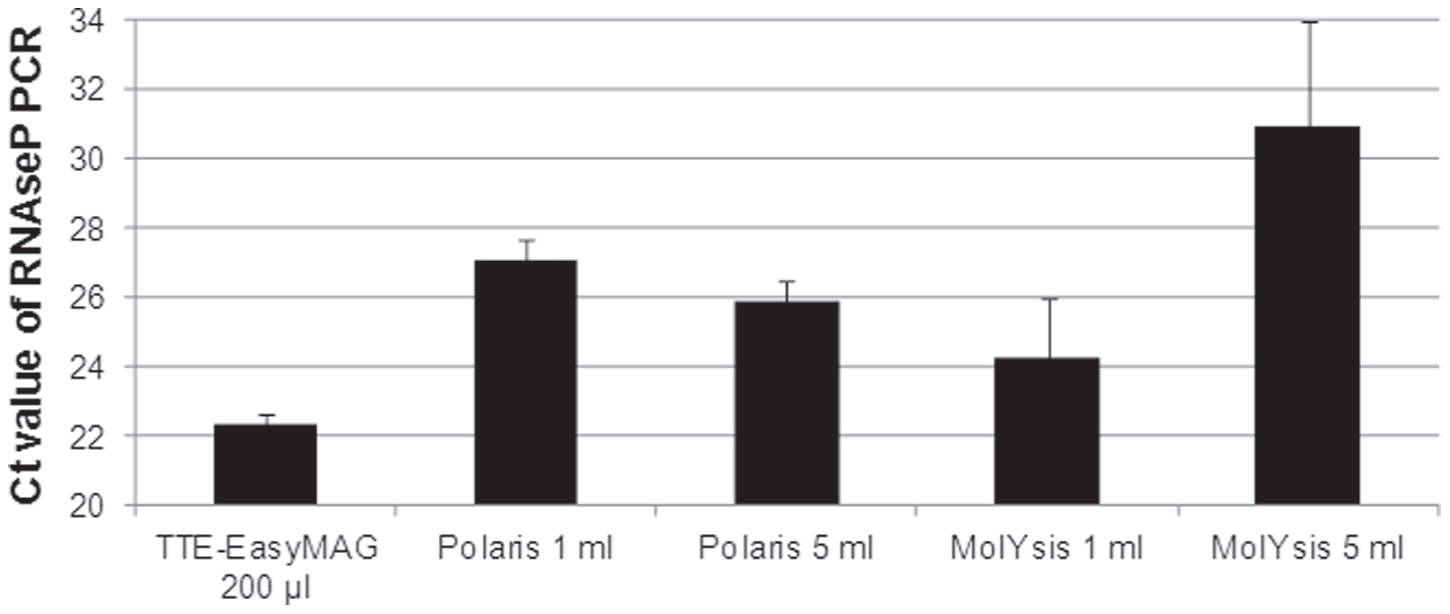
Human DNA removal by different procedures. Ct value comparison of the RNAseP PCR for all pathogen DNA isolation procedures. RNAseP is a marker to measure human DNA removal after pathogen DNA isolation. Standard deviations are shown of at least 6 independent experiments. One-way ANOVA analysis indicated that TTE-EasyMAG removes the least amount of human DNA as compared to all other methods (*p*<0.001), except when compared to MolYsis 1 ml (*p = *0.156). MolYsis 5 ml removes most human DNA as compared to all other methods (*p*<0.000). Significant differences were also found when comparing Polaris 1 ml with both MolYsis 1 ml (*p = *0.002) and 5 ml (*p*<0.000), and when comparing MolYsis 1 ml with MolYsis 5 ml (*p*<0.000).

To assess human DNA removal capacity of the different methods, an RNAseP PCR was performed. The obtained Ct-values (Figure 5) show that the TTE-EasyMAG method removes the least amount of human DNA (lowest Ct-value for RNAseP). One-way ANOVA analysis indicated that this was statistically significant to all other methods (*p*<0.001), except when compared to MolYsis 1 ml (*p = *0.156). The MolYsis method for 5 ml of whole blood removes most human DNA as compared to all other methods (*p*<0.000). Significant differences were also found when comparing Polaris for 1 ml whole blood with both MolYsis for 1 ml (*p = *0.002) and the MolYsis method for 5 ml whole blood (*p*<0.000), and when comparing MolYsis for 1 ml with MolYsis for 5 ml whole blood (*p*<0.000). No significant difference in human DNA removal capacity was found between the Polaris methods for different volumes of whole blood (*p = *0.548).

The lower Ct value for RNAseP in Figure 5 showed that the amount of residual human DNA was higher in the 1 ml MolYsis protocol than in the 5 ml MolYsis protocol. For the 1 ml protocol the volume of blood in the total lysate is 1/1.5 or 66%, whereas in the 5 ml protocol this is 5/9 or 55%. In comparison, the 1 ml Polaris protocol was more efficient in DNA removal than the 1 ml MolYsis protocol, whereas for the 5 ml protocols (MolYsis versus Polaris) it was the other way around. However, both the MolYsis and the Polaris method removed sufficient human background so no interference with the specific pathogen PCR was detected.

### Comparison of Polaris, TTE-EasyMAG and MolYsis

The TTE-EasyMAG procedure yielded higher Ct values for most samples compared to the Polaris samples (up to 6 Ct difference). Polaris and MolYsis resulted in comparable Ct values for all pathogens (Figure 3). Both MolYsis and Polaris enabled detection of clinical relevant pathogen concentrations of 1–10 CFU/ml. In general, the variation in Ct values was much larger for MolYsis-processed samples than for samples processed with Polaris (Figure 3).

Calculations of detection rates, i.e. percentages of positive PCRs, demonstrated a detection rate of 100% for all pathogens at a concentration of 10 CFU/ml for the Polaris procedure (Table 2). The TTE-EasyMAG procedure performed much worse in this respect with a detection rate of only 58%, 60%, and 79% for 10 CFU/ml *S. aureus*, *C. albicans,* and *P. aeruginosa,* respectively. MolYsis resulted in a detection rate of 50%, 67%, and 58% for 10 CFU/ml *S. aureus*, *C. albicans,* and *P. aeruginosa,* respectively. Processing samples containing 1 CFU/ml never resulted in a 100% detection rate for the tested methods. The best results were obtained with Polaris as a 70% detection rate was obtained for *S. aureus*, and 75% for both *C. albicans* and *P. aeruginosa.* MolYsis detection rates at this pathogen concentration varied between 17 and 50%, and TTE-EasyMAG between 20–36%. All non-spiked blood samples were negative in the PCRs.

**Table 2:** Detection rates (percentage of positive PCRs) of 3 different DNA isolation methods in dilutions series of 1–1000 CFU/ml.

		CFU/ml				
		1000	100	10	1	*p* ^a,b,c^
***P. aeruginosa***	Polaris 5 ml	100% (12/12)	100% (12/12)	100% (12/12)	75% (9/12)	a (*p* = 0.01)
	MolYsis 5 ml	83% (10/12)	92% (11/12)	58% (7/12)	17% (2/12)	b (*p = *0.06)
	TTE-EasyMAG	100% (8/8)	92% (11/12)	79% (11/14)	36% (5/14)	c (*p* = 0.39)
***C. albicans***	Polaris 5 ml	100% (12/12)	83% (10/12)	100% (12/12)	75% (9/12)	a (*p* = 0.04)
	MolYsis 5 ml	92% (11/12)	83% (10/12)	67% (8/12)	25% (3/12)	b (*p* = 0.08)
	TTE-EasyMAG	100% (10/10)	100% (10/10)	60% (6/10)	30% (3/10)	c (*p* = 1.00)
***S. aureus***	Polaris 5 ml	100% (6/6)	100% (10/10)	100% (12/12)	70% (7/10)	a (*p* = 0.63)
	MolYsis 5 ml	100% (8/8)	75% (6/8)	50% (4/8)	50% (4/8)	b (*p* = 0.07)
	TTE-EasyMAG	100% (6/6)	90% (9/10)	58% (7/12)	20% (2/10)	c (*p* = 0.32)

Fisher’s exact test performed on 1 CFU/ml samples, statistically significant when *p*≤0.05. a; Polaris versus MolYsis, b; Polaris versus TTE-EasyMAG, c; MolYsis versus TTE-EasyMAG.

The Fisher’s exact test was performed to show significant differences between the obtained detection rates (1 CFU/ml) with the different pathogen DNA isolation methods for each pathogen. There was never a significant difference found, in detection rate, when comparing TTE-EasyMAG with MolYsis (5 ml) or Polaris (5 ml) for all tested pathogens. However, TTE-EasyMAG compared with Polaris showed to have lower *p*-values (*p* between 0.06–0.08) as when compared with MolYsis (*p* between 0.32–1.00). For *P. aeruginosa,* Polaris had a significant better detection rate when compared to MolYsis (*p = *0.01). This difference was also seen for *C. albicans* (*p = *0.04). No significant difference in detection rate for *S. aureus* was found between Polaris and MolYsis (*p = *0.63).

## Discussion

In this study, different pathogen DNA isolation methods for whole blood were compared. We showed that both MolYsis and Polaris enrichment followed by DNA isolation and real-time PCR enabled reliable and sensitive detection of bacteria and fungi from 5 ml blood. MolYsis and the TTE-EasyMAG procedure resulted in a lower number of positive PCRs (detection rate) as compared to Polaris, especially in the lower limit of detection (1–10 CFU/ml).

The detection rates for *P. aeruginosa* and *S. aureus* detection are similarly high at a pathogen concentration of 1 CFU/ml when using Polaris (9/12 vs. 7/10) (Table 2). In contrast, using MolYsis enrichment, the detection rate for *P. aeruginosa* is considerably lower than that for Gram-positive *S. aureus* detection (2/12 vs. 4/8). Possibly, Gram-negative bacteria which generally are considered to be more fragile than Gram-positives may be negatively affected by the chaotropic buffer used in the MolYsis protocol to lyse human cells [18]. Furthermore, the Ct values obtained in the *C. albicans* PCR are lower compared to Ct values obtained in the other PCRs. This might be the result of copy number variations (5 versus single copy) [19]. However, the detection limits of all PCRs are similar.

Human DNA, which can interfere in the PCR reaction, was not removed when the TTE-EasyMAG procedure was used. In contrast, Polaris and MolYsis enrichment resulted in substantial removal of human DNA as was shown by the RNAseP results and the fact that the reference and whole blood samples showed similar Ct values. There are differences in the ratio of blood and lysis buffer volumes between the 1 and 5 ml MolYsis protocols. It was noticed that when using MolYsis the 1 ml blood lysates were much more viscous than the 5 ml lysates. Apparently, DNAse treatment is much less efficient in the more viscous 1 ml lysate. This might also explain the high variability in residual human DNA levels in the 1 ml MolYsis protocol.

In general, Ct values obtained after Polaris processing were much more constant than those after MolYsis processing. Several steps in the MolYsis procedures may contribute to this variation. Next to the chaotropic buffer mentioned above, the use of an enzyme to degrade DNA may yield variable results, due to enzyme instability. Furthermore, bacterial lysis is based on a mix of lytic enzymes and proteinase K. The Polaris procedure does not use chaotropic agents nor enzymes, but only chemicals that should remain stable over time. Furthermore, it was demonstrated that Polaris pathogen enrichment can be combined with both QIAamp and EasyMAG (generic) DNA purification. The preliminary data showed that Polaris combined with EasyMAG DNA purification holds most promise to obtain reliable data at borderline concentrations of 1 CFU/ml. The benefit of using more concentrated DNA as input (EasyMAG) in the PCR was not negatively affected by concurrent concentration of inhibitory substances.

Polaris and MolYsis have shown to be valuable in spiked blood samples since they can handle large blood volumes. Clinical evaluation of Polaris is presently ongoing in comparison to MolYsis, which is clinically validated. Preliminary results of this ongoing study (Emergency Care Unit, Jeroen Bosch Hospital) show that both pathogen enrichment procedures work for clinical samples. Residual blood was collected, left over from standard diagnostics, from patients with bloodcultures positive for *S. aureus* (1 culture) or *S. pneumoniae* (2 cultures) (data not shown). This approach limited the volume of usable blood to 1 ml for each method. Polaris was followed by DNA purification using EasyMAG. We were able to detect *S. pneumoniae* and *S. aureus* in all 3 samples with corresponding positive blood cultures, indicating promising potential for both the Polaris and MolYsis procedure in clinical use.

Several molecular sepsis diagnosis tests have become commercially available recently, i.e. Roche’s Septi*FAST*, Seegene’s MagicPlex Sepsis Real-Time Test, VYOO (SIRS lab) and Molzym’s SepsiTest. It has been shown, by independent research groups, that these diagnostic tests are complementing conventional culture techniques [12], [13], [20], [21]. Especially in antibiotic-treated patients, molecular diagnostics can provide identification under conditions where blood cultures remain negative. Recent publications by Kühn and Wellinghausen [11], [12] show the value of Molzym’s SepsiTest. Both described that the initial analysis, indicating the absence or presence of pathogens, can be performed in approximately 4 hours. Subsequent sequencing needs to be performed for specific pathogen identification. This approach has the advantage that any pathogen will be identified, but it takes an additional 4 hours (in an optimal setting) to obtain that result. Still, pathogen identification is available within one working day, which is faster compared to conventional culture techniques that take at least 24–72 hours for pathogen identification. Assays like Septi*FAST* and Seegene’s MagicPlex Sepsis Test have other limitations. In the Septi*FAST* procedure no enrichment of pathogen DNA is included, this limits the maximal useable input blood volume to 1.5 ml with an equivalent of only 0.167 ml blood present in the PCR reaction. The detection system used in the Septi*FAST* method enables rapid identification of 25 pathogens by multiplex real-time PCR followed by melting curve analysis. Seegene’s MagicPlex real-time PCR test can be used in combination with MolYsis pathogen enrichment. The real-time PCR test enables the detection of 90 BSI causing pathogens, but only 27 pathogens can be identified to the species level. The main disadvantage of the Seegene system is the fact that one first needs to create an amplicon bank via conventional PCR. Next, the vial containing PCR amplicons needs to be opened for subsequent signal amplification in a real-time PCR instrument. Most routine diagnostic laboratories would not allow this setup, as contamination risks exist. Polaris enrichment can be combined with established sepsis tests to be able to ensure broad pathogen detection from clinical samples.

In conclusion, Polaris and MolYsis enrichment followed by DNA isolation and real-time PCR enables reliable and sensitive detection of bacteria and fungi from 5 ml blood. However, Polaris is slightly more sensitive and faster providing pathogen identification within 3 hours. To further enable its clinical value, Polaris is currently being automated in a closed disposable cartridge to reduce the hands on time to 1–2 min, to be faster, and less prone to contamination. Furthermore, the combination of the Polaris cartridge with commercially available sepsis tests is currently being evaluated in a prospective clinical study using 5 ml whole blood.
